# Conditional Deletion of the *Phd2* Gene in Articular Chondrocytes Accelerates Differentiation and Reduces Articular Cartilage Thickness

**DOI:** 10.1038/srep45408

**Published:** 2017-03-28

**Authors:** Shaohong Cheng, Sheila Pourteymoor, Catrina Alarcon, Subburaman Mohan

**Affiliations:** 1 Musculoskeletal Disease Center, Veterans Affairs Loma Linda Healthcare System, 11201 Benton Street, Loma Linda, CA 92357, USA; 2 Department of Medicine, Loma Linda University, Loma Linda, CA 92354, USA.

## Abstract

Based on our findings that PHD2 is a negative regulator of chondrocyte differentiation and that hypoxia signaling is implicated in the pathogenesis of osteoarthritis, we investigated the consequence of disruption of the *Phd2* gene in chondrocytes on the articular cartilage phenotype in mice. Immunohistochemistry detected high expression of PHD2 in the superficial zone (SZ), while PHD3 and HIF-1α (target of PHD2) are mainly expressed in the middle-deep zone (MDZ). Conditional deletion of the *Phd2* gene (cKO) in chondrocytes accelerated the transition of progenitors to hypertrophic (differentiating) chondrocytes as revealed by reduced SZ thickness, and increased MDZ thickness, as well as increased chondrocyte hypertrophy. Immunohistochemistry further revealed decreased levels of progenitor markers but increased levels of hypertrophy markers in the articular cartilage of the cKO mice. Treatment of primary articular chondrocytes, *in vitro*, with IOX2, a specific inhibitor of PHD2, promoted articular chondrocyte differentiation. Knockdown of *Hif-1α* expression in primary articular chondrocytes using lentiviral vectors containing *Hif-1α* shRNA resulted in reduced expression levels of *Vegf, Glut1, Pgk1*, and *Col10* compared to control shRNA. We conclude that *Phd2* is a key regulator of articular cartilage development that acts by inhibiting the differentiation of articular cartilage progenitors via modulating HIF-1α signaling.

Articular cartilage is a hyaline cartilage that provides a smooth and frictionless surface for bone movements within a joint. The thin layer of articular cartilage covers the bone surface and maintains its lubricating function for an individual’s lifetime. Damage to the articular cartilage is a significant clinical issue causing an enormous economic burden worldwide. The most common articular cartilage damage is osteoarthritis characterized by progressive degeneration of articular cartilage that leads to chronic pain and functional restrictions in the affected joints. Pathophysiological features of osteoarthritis include degeneration or loss of articular cartilage, sclerosis, an increase of subchondral bone density, and osteophyte formation^1^,^2^,^3^. The precise mechanisms that regulate the integrity of articular cartilage and the mechanisms of osteoarthritis development are still unclear and nonsurgical therapeutic interventions to articular cartilage diseases are limited to date^3^,^4^.

The articular cartilage is composed of several chondrocyte layers with unique cellular arrangements including a superficial zone (SZ), a middle zone, a deep zone, and a calcified cartilage zone. The SZ contains flat shaped articular cartilage progenitor cells robustly expressing proteoglycans such as aggrecan and lubricin^5^,^6^, while chondrocytes in the deep zone are in a columnar organization with some of them becoming hypertrophic and marked by expression of *Col10*^2^. A number of growth factors including Wnt9A, TGFα, PTHrP, IGF-I and BMPs have been implicated in maintaining articular cartilage integrity by regulating the transit of articular cartilage progenitors towards differentiating chondrocytes^6^,^7^,^8^. It is known that increased chondrocyte hypertrophy, which leads to increased endochondral ossification, is frequently identified in early stage osteoarthritis samples^9^,^10^. Accordingly, studies have shown an inverse correlation between the onset of osteoarthritis and an increase in subchondral bone density^11^,^12^. Hence, understanding the molecular mechanisms controlling the transition of articular cartilage progenitors towards hypertrophic chondrocytes is important for the prevention and treatment of articular cartilage diseases.

Prolyl Hydroxylase Domain-containing Proteins (PHDs) are negative regulators of the hypoxia-inducible factors (HIFs) which moderate chondrocyte differentiation^13^,^14^,^15^. PHDs belong to the 2-oxoglutarate/iron-dependent dioxygenase superfamily. There are three members of *Phds* in mammals: *Phd1 Phd2,* and *Phd3.* All PHDs contain the highly conserved hydroxylase domain in the catalytic carboxy-terminal^16^. PHD2 is the major regulator of HIF-1α activity. In the presence of oxygen, PHD2 hydroxylates two proline residues (Pro-402 and Pro-564) in the C-terminal of HIF-1α which leads to the ubiquitin-mediated proteasomal degradation of HIF-1α^17^,^18^. HIF-1α is required for the maintenance and differentiation of chondrocytes in the hypoxic growth plate^19^. Conditional deletion of HIF-1α in mesenchymal cells resulted in abnormal cartilage and joint development^20^,^21^. On the other hand, we found that deletion of *Phd2* in chondrocytes caused accelerated chondrocyte differentiation and increased endochondral bone formation in the metaphyses and epiphyses of long bones^13^,^14^. These data clearly demonstrate that *Phd2* negatively regulates chondrocyte differentiation and endochondral bone formation through the PHD/HIF regulatory pathway.

Interestingly, in addition to the growth plate chondrocytes, *Phd2* and *Phd3* are also highly expressed the articular chondrocytes with distinct expression patterns. Since *Phd2* is a negative regulator of chondrocyte differentiation, we hypothesize that *Phd2* also inhibits the differentiation of articular cartilage progenitors, and deletion of *Phd2* in chondrocytes promotes progenitors to differentiate into hypertrophic chondrocytes and thereby, reduce articular cartilage thickness. Since articular cartilage richly contains Type II collagen (*Col2*), we used *Col2α1-Cre* line to conditionally delete *Phd2* gene in articular chondrocytes.

## Results

### PHDs and HIFs are expressed in distinct patterns in the femoral articular cartilage

**Figure 1:**
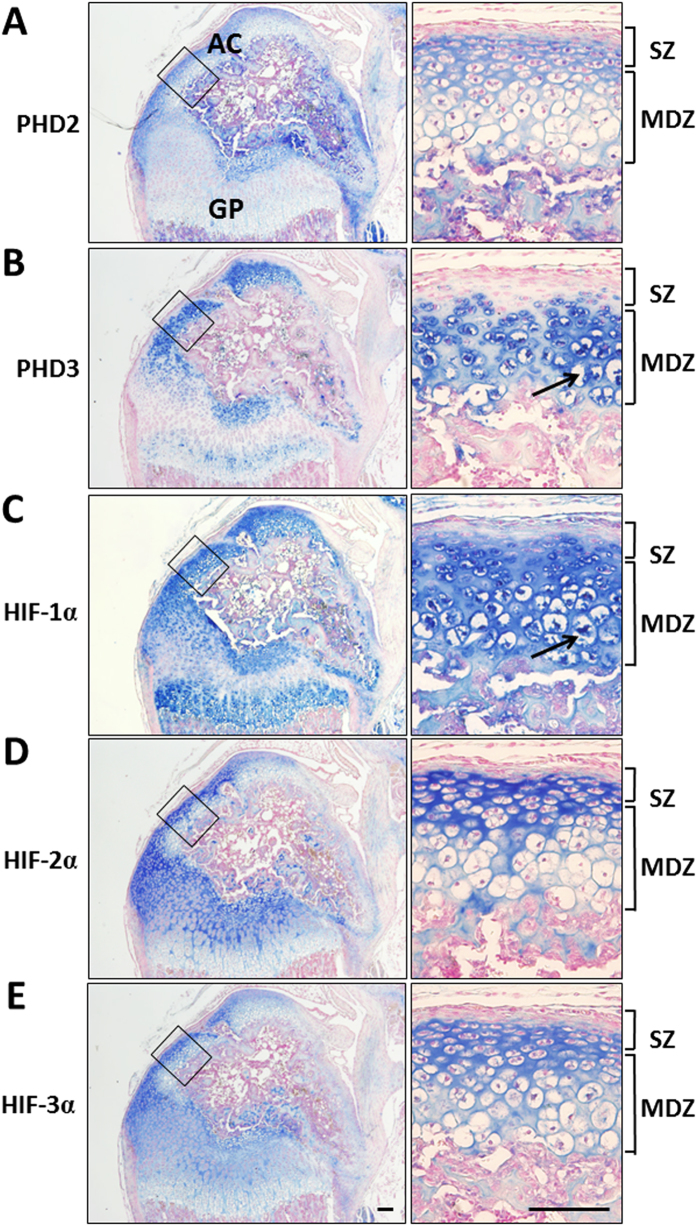
Expression patterns of PHDs and HIFs in the articular cartilage. Immunohistochemistry was performed on femur sections from 2 week old mice. Left panels show expression of each protein in whole femur heads and right panels are high magnifications of the articular cartilage. (**A**) PHD2 is highly expressed in the SZ, but remains low in the MDZ. (**B**) PHD3 is highly expressed in the MDZ, but remains low in the SZ. (**C**) HIF-1α protein is highly expressed in the MDZ. An arrow shows a hypertrophic chondrocyte. (**D**) HIF-2α protein is highly expressed in the SZ. (**E**) HIF-3α is ubiquitously expressed in the articular cartilage. Blue is positive staining. AC, articular cartilage; GP, growth plate; SZ, superficial zone; MDZ, middle-deep zone. Bar = 50 μm. Magnification: left panel, 40X; right panel, 200X.

To study the role of PHDs in articular cartilage development, we first evaluated the expression patterns of PHD2 and PHD3 and their targets (HIFs) using immunohistochemistry in the distal femoral articular cartilage in 2 week old mice, when articular cartilage formation is known to occur. PHD2 protein is highly expressed in the SZ of articular cartilage, but remains low in the middle-deep zone (MDZ) (Fig. 1A, blue is positive staining). By contrast, PHD3 is almost absent in the SZ, but highly expressed in the MDZ, where some of the chondrocytes are undergoing hypertrophy (Fig. 1B). HIF-1α protein, a known target of PHD2, appears mainly in the MDZ of articular cartilage, whereas PHD2 expression is low (Fig. 1C). On the other hand, HIF-2α is highly expressed in the SZ, but not in the MDZ of the articular cartilage, very similar to the expression pattern of PHD2 (Fig. 1D). HIF-3α is more ubiquitous than other HIFs and is expressed throughout different zones at relatively low levels (Fig. 1E). The high expression of *Phd2* and *Hif-2α* in the progenitor containing SZ suggests a role for *Phd2* and *Hif-2α* in maintaining the progenitor status of articular chondrocytes. On the other hand, the predominant expression of *Phd3* and *Hif-1α* in MDZ implicates their role in articular chondrocyte differentiation.

### Conditional deletion of *Phd2* in the *Col2*-expressing cells ablated PHD2 expression in articular cartilage

**Figure 2:**
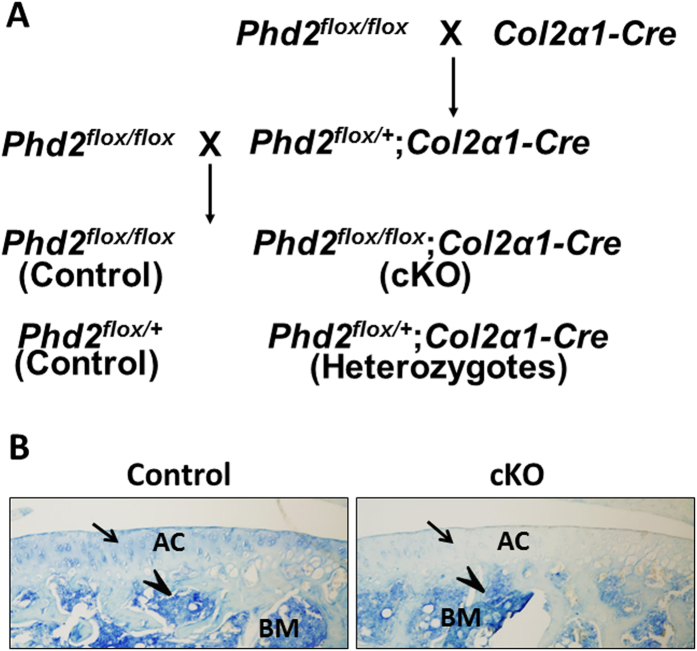
Generating the conditional *Phd2* knockout mice in articular chondrocytes. (**A**) Conditional deletion of the *Phd2* gene in *Col2α1*-expressing chondrocytes. (**B**) Immunohistochemistry analysis of PHD2 expression in tibiae of 4 week old control and cKO mice. PHD2 was expressed in the control but not in the cKO tibial articular cartilage (arrows). Expression of PHD2 in bone marrow was intact in the cKO mice (arrow heads). Blue is positive staining. BM, bone marrow.

To test the hypothesis that *Phd2* plays a role in maintaining the progenitor status of the cells in the SZ by preventing their differentiation towards a hypertrophic stage, we used *Col2α1-Cre* transgenic mice to disrupt the *Phd2* gene in articular chondrocyte progenitors that express high levels of *Col2α1* (Fig. 2A). In skeletal tissue, *Col2α1-Cre* activity was specifically detected in chondrocytes and the specificity of the *Col2α1-Cre* line was documented in several studies^22^,^23^,^24^,^25^. We have also used this line to knockout *Phd2* in chondrocytes to study *Phd2* function in endochondral bone formation at the primary and secondary ossification centers^13^,^14^. The efficiency of the *Col2α1-Cre* line used for disruption of the *Phd2* gene in articular chondrocytes was validated by immunohistochemistry. In Fig. 2B, PHD2 protein was detected in tibial articular cartilage of 4 week old control mice, but not in the conditional knockout (cKO) mice (Fig. 2B, blue is positive staining). By contrast, PHD2 protein expressed in bone marrow was intact in the cKO mice compared to the control mice (Fig. 2B).

### Deletion of *Phd2* in articular cartilage reduced the thickness of the superficial zone and increased chondrocyte hypertrophy in articular cartilage

**Figure 3:**
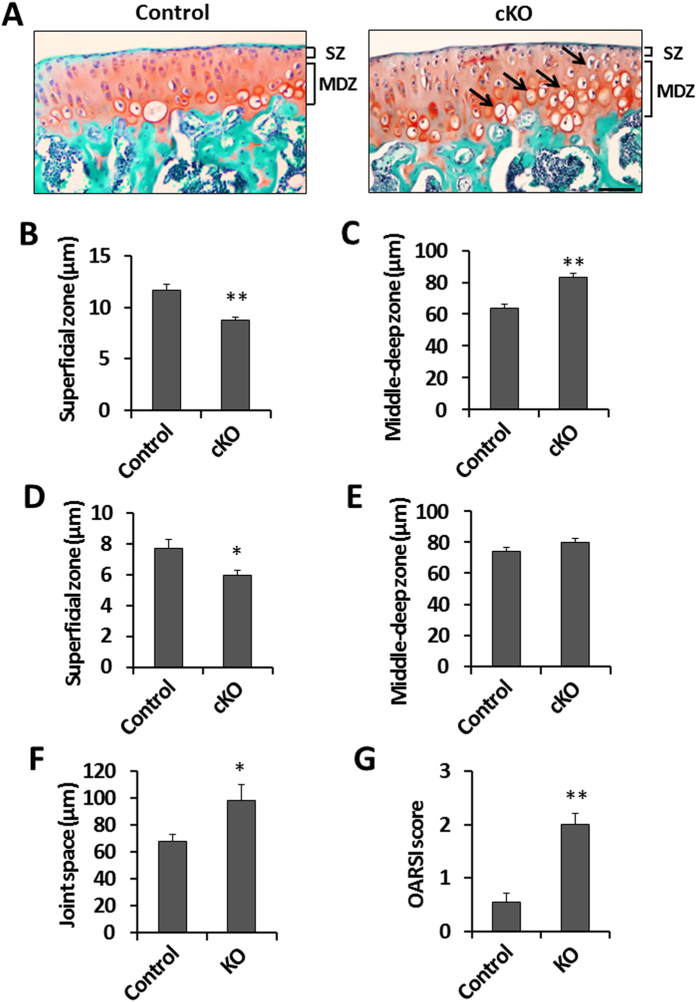
Histological changes in the articular cartilage of the *Phd2* cKO mice. (**A**) Safranin-O staining of 4 week old control and cKO tibiae. Arrows show hypertrophic chondrocytes. (**B**) SZ thickness in control and cKO tibiae at 4 weeks of age. (**C**) MDZ thickness in control and cKO tibiae from 4 week old mice. (**D**) SZ thickness in 12 week old control and cKO tibiae. (**E**) MDZ thickness in 12 week old control and cKO tibiae. (**F**) Thickness of joint space in 12 week old control and cKO tibiae. (**G**) OARSI score in 12 week old control and cKO tibiae. *P <* *0.05, **P <* *0.01, *n *=* *8/group in (**B,C**), *n *=* *6/group in (**D**–**G**). Data were presented as the mean ± SEM. Bar = 50 μm.

To evaluate the effect of *Phd2* deletion on articular cartilage development, the articular cartilage phenotype was evaluated by histology in the long bones of 4 week old mice. We measured the thickness of the SZ and MDZ in the tibial articular cartilage. The thickness of SZ was reduced by 25% (*P* < 0.05) in the cKO tibia compared to control tibia (Fig. 3A,B). By contrast, the thickness of MDZ was increased by 31% (*P* < 0.05) in the cKO tibia compared to controls (Fig. 3A,C). The number of hypertrophic chondrocytes was increased in the articular cartilage of the cKO mice compared to controls (Fig. 3A), indicating increased chondrocyte differentiation in the articular cartilage of the cKO mice. These data suggests that loss of *Phd2* promoted the transition of articular cartilage progenitors in the SZ into differentiating chondrocytes.

To examine if the histological changes of the articular cartilage in the cKO mice were maintained in adult mice, we also measured the thickness of the SZ and the MDZ in 12 week old mice. The reduction in SZ thickness in the cKO tibia was 23% (*P* < 0.05) compared to control tibia in 12 week old mice (Fig. 3D). The thickness of the MDZ was slightly increased in the cKO mice compared to control mice, but this change did not reach statistical significance (p = 0.19) (Fig. 3E). We further evaluated an early stage osteoarthritis phenotype in these mice. We found that the joint space was increased by 44% (*P* < 0.05), and the Osteoarthritis Research Society International (OARSI) score was increased by 270% (*P* < 0.01) in the cKO mice compared to control mice (Fig. 3F,G), suggesting that a lack of *Phd2* may increase the risk of osteoarthritis development.

### Deletion of *Phd2* in articular cartilage reduced expression of progenitor markers, but elevated expression of hypertrophy markers

**Figure 4:**
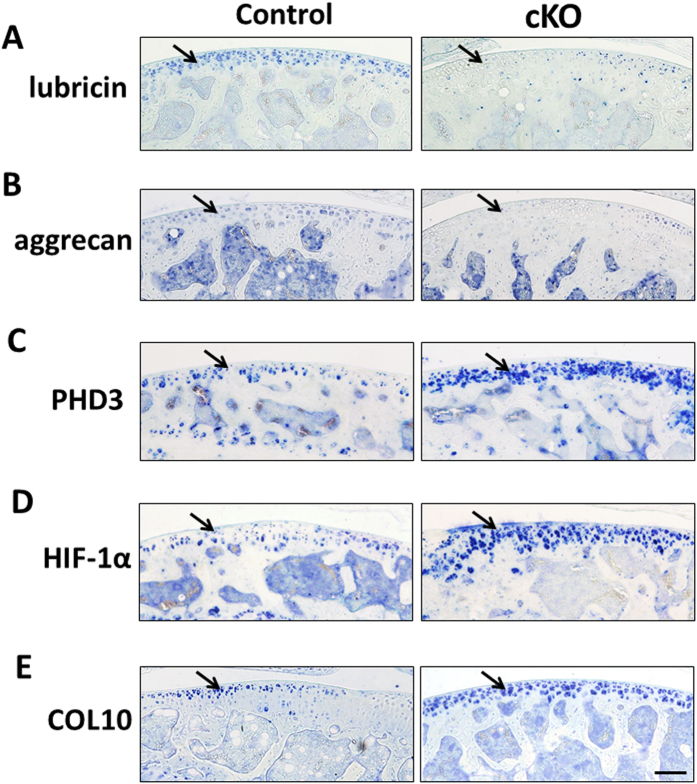
Expression of markers of articular cartilage progenitors and chondrocyte differentiation in control and cKO mice. Immunohistochemistry analysis of markers in tibial articular cartilage of 12 week old control and cKO mice. (**A**,**B**) Expression of SZ progenitor markers, lubricin and aggrecan, in articular cartilage. (**C**) PHD3 expression in control and cKO articular cartilage. (**D**) HIF-1α expression in control and cKO articular cartilage. (**E**) Expression of the chondrocyte hypertrophy marker COL10 in control and cKO articular cartilage. Blue is positive staining. Arrows show positive stains of each protein in the articular cartilage. Bar = 50 μm.

Articular cartilage progenitors in the SZ express high levels of lubricin and aggrecan^5^,^6^. Consistent with the prediction that loss of *Phd2* promoted chondrocyte differentiation, we found expression levels of both lubricin and aggrecan were markedly reduced in the cKO articular cartilage (Fig. 4A,B, blue is positive staining) while levels of PHD3 and HIF-1α, that are known to be highly expressed in hypertrophic chondrocytes, were elevated in the cKO articular cartilage compared to controls (Fig. 4C,D). We also examined the expression of COL10, a well-established marker of hypertrophic chondrocytes, and found that it was markedly increased in the cKO articular chondrocytes (Fig. 4E), thus suggesting elevated chondrocyte differentiation in the articular cartilage of the *Phd2* cKO mice.

### Inhibition of PHD2 by IOX2 promoted cell differentiation and HIF-1α signaling in primary articular chondrocytes

**Figure 5:**
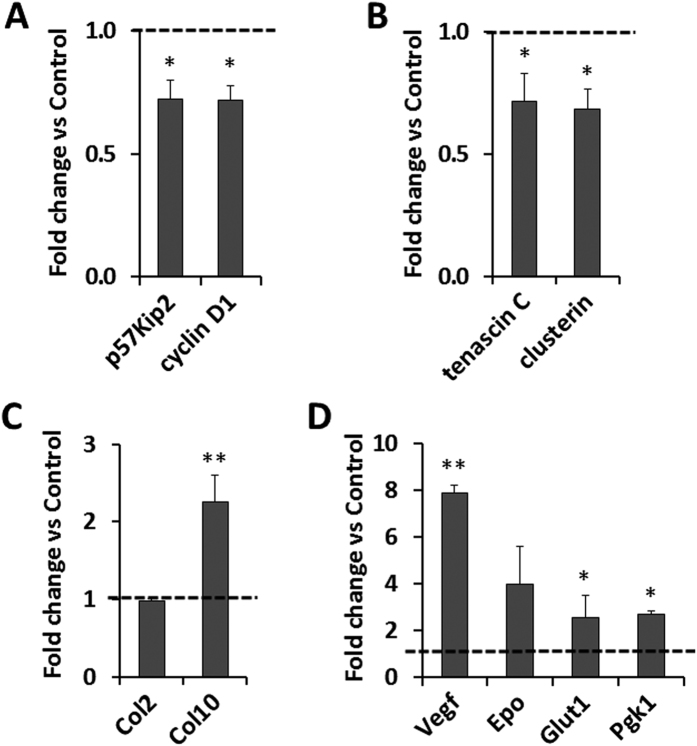
IOX2-mediated inhibition of PHD2 promotes differentiation of articular chondrocytes. Primary articular chondrocytes were cultured with 20 μM PHD2 inhibitor IOX2 or vehicle (DMSO) for 72 hours and expression of markers were measured by Real time RT-PCR. (**A**) mRNA expression of cell proliferation marker p57Kip2 and cyclin D1. (**B**) mRNA expression of SZ progenitor marker tenascin C and clusterin. (**C**) mRNA expression of chondrocyte marker *Col2* and chondrocyte hypertrophy marker *Col10*. (**D**) mRNA expression of HIF-1α targets, vascularization marker *Vegf, Epo*, and glycolysis marker *Glut1* and *Pgk1*. *P <* *0.05, **P <* *0.01, *n *=* *4/group. Data were normalized to controls and presented as the mean ± SEM.

In order to determine if the increased differentiation of articular chondrocytes in the *Phd2* cKO mice is a direct consequence of loss of PHD2 function, we treated primary articular chondrocytes with a specific inhibitor of PHD2, IOX2, for 72 hours and then measured expression levels of markers of proliferation, articular cartilage progenitors, and chondrocyte differentiation. We found that expression of proliferation markers, p57 and cyclin D1, were both reduced by 28% (*P *<* *0.05) (Fig. 5A). Accordingly, expression levels of markers of articular cartilage progenitors, tenascin c and clusterin, were reduced by 28% and 32%, respectively (*P *<* *0.05) (Fig. 5B). Expression of *Col2* was unchanged (Fig. 5C). By contrast, expression level of *Col10* was increased by 126% (*P *<* *0.01) (Fig. 5C). Loss of PHD2 activity is known to increase protein levels of HIF-1α via inhibition of prolyl hydroxylation of HIF-1α and its subsequent proteosomal degradation^17^,^18^. Activation of HIF-1α signaling is known to promote angiogenesis and glycolysis^26^,^27^,^28^. Accordingly, expression levels of *Vegf*, an angiogenic marker and a known target of HIF-1α, were increased by 8 fold (*P *<* *0.01) (Fig. 5D). *Epo* is also a target of HIF-1α. Its expression was also increased, but the increase did not reach statistical significance. Expression levels of *Glut1* and *Pgk1*, markers of glycolysis, were increased by 154% and 170%, respectively (*P *<* *0.05) in IOX2 treated cells (Fig. 5D).

### Knockdown of *Hif-1α* reduced differentiation and glycolysis of primary articular chondrocytes

**Figure 6:**
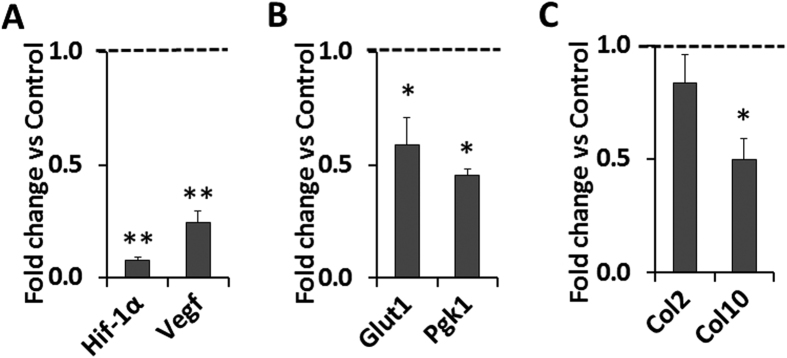
shRNA knockdown of HIF-1α reduced expression of markers of glycolysis and chondrocyte differentiation. Primary articular chondrocytes were treated with lentiviral vectors containing shRNA specific to *Hif-1α* or control shRNA. (**A**) *Hif-1α* mRNA expression was knocked down by 92%. *Vegf* mRNA expression was significantly reduced in *Hif-1α* knockdown cells. (**B**) mRNA Expression of glycolysis marker *Glut1* and *Pgk1* in the *Hif-1α* knockdown cells. (**C**) mRNA expression of *Col2* and *Col10* in the *Hif-1α* knockdown cells. *P <* *0.05, **P <* *0.01, *n *=* *3/group. Data were normalized to control shRNA treated cells and presented as the mean ± SEM.

In order to determine the role of HIF-1α signaling in mediating *Phd2* effects on chondrocyte differentiation, we evaluated the consequence of knockdown of *Hif-1α* in primary articular chondrocytes. We used lentiviral vectors containing shRNA specific to *Hif-1α* to knockdown HIF-1α. *Hif-1α* shRNA treatment knocked down *Hif-1α* mRNA expression by 92% (*P *<* *0.01) compared to control shRNA (Fig. 6A). Expression of the HIF-1α target, *Vegf*, was also reduced by 75% (*P *<* *0.01) (Fig. 6A). Furthermore, HIF-1α targets, the glycolytic enzymes *Glut1* and *Pgk1*, were reduced by 41% and 55%, respectively (*P *<* *0.05) (Fig. 6B). The chondrocyte hypertrophy marker *Col10* was also reduced by 50% (*P *<* *0.05), while *Col2* expression was not changed (Fig. 6C). The findings that knockdown of *Hif-1α* inhibited angiogenesis, glycolysis, and chondrocyte differentiation, effects that are opposite to those of IOX2 mediated inhibition of PHD2 activity, suggests that the negative effect of *Phd2* on chondrocyte differentiation in articular cartilage is perhaps mediated via inhibition of the HIF-1α signaling pathway.

## Discussion

In this study, we have found distinct spatial distribution of PHD2 and PHD3 in the articular cartilage, with PHD2 being mainly localized to the SZ progenitor cells while PHD3 to the differentiating chondrocytes in the MDZ. The low level of PHD2 in the MDZ was accompanied by a high level of HIF-1α in the MDZ, consistent with the well-known function of PHD2 to target HIF-1α to proteosomal-mediated degradation^17^,^18^. Conditional deletion of *Phd2* in chondrocytes decreased the thickness of progenitors in the SZ and increased chondrocyte hypertrophy in the MDZ of the tibial articular cartilage, marked by down regulation of lubricin and aggrecan, and up regulation of HIF-1α and COL10. These findings suggest that high levels of *Phd2* are required to maintain the progenitor status of cells in the SZ. Loss of functional PHD2 in the progenitor cells of the SZ promotes articular chondrocyte differentiation. This notion is further supported by a primary articular chondrocyte study showing that inhibition of PHD2 suppressed expression of tenascin C and clusterin, but elevated expression of *Col10* and HIF-1α. The *Phd2* effect on articular chondrocyte differentiation appears to be mainly mediated by HIF-1α signaling as revealed from our data from experiments involving knockdown of *Hif-1α* and PHD2 inhibitor.

Our study demonstrated a crucial role for *Phd2* in articular chondrocyte regulation. Similar to its role in endochondral bone formation, *Phd2* inhibits HIF-1α signaling and hence inhibits chondrocyte differentiation^13^,^14^. It is well established that HIF-1α is a positive regulator of chondrocyte differentiation and ossification, as conditional deletion of the von Hippel-Lindau gene (Vhl), a ubiquitin ligase that promotes proteolysis of HIFs, or overexpression of *Vegf*, a HIF-1α target, resulted in excessive endochondral bone formation^29^,^30^. HIF-1α signaling also appeared to negatively affect progenitor status of the articular chondrocytes, as Phd2 deletion in chondrocytes resulted in reduced articular cartilage SZ thickness. The negative regulation of HIF-1α signaling in progenitors was also supported by the *Hif-1α* knockdown experiment. *Hif-1α* knockdown reduced expression of markers of proliferation, as well as markers of progenitors. Based on these data, one would expect a negative effect of HIF-1α on the maintainence of articular cartilage, and higher levels of HIF-1α could contribute to the pathogenesis of osteoarthritis.

In addition to its established role in regulating chondrocyte differentiation, other studies have shown that *Hif-1α* is also required for chondrocyte growth arrest and survival. Lack of *Hif-1α* resulted in chondrocyte death^19^. Balb/C mice injected with HIF-1α inhibitor, 2-methoxyestradiol, resulted in progressive destruction of the articular cartilage, and HIF-1α inhibition in human osteoarthritic cartilage accelerates catabolic stress-induced apoptosis *in vitro*, thus suggesting a protective role for *Hif-1α* in osteoarthritis development^31^,^32^. However, this hypothesis contradicted research showing that stabilization of HIF-1α by dimethyloxaloylglycine did not prevent osteoarthritis development in the knee joints of STR/ORT mice^31^. Nevertheless, *Hif-1α* appeared to be clinically important in the development of osteoarthritis. Several studies reported elevation of HIF-1α signaling in human osteoarthritic samples^32^,^33^,^34^. The precise role of HIF-1α in articular cartilage development and the clinical significance of aberrant PHD2/HIF-1α signaling in osteoarthritis development needs to be investigated further.

Though PHD2 is the major regulator of HIF-1α, HIF-1α also interacts with PHD3. HIF-1α up regulates PHD3 through a feedback mechanism^14^. On the other hand, PHD3 hydroxylates HIF-1α, although PHD3 is the major regulator of HIF-2α^35^. In terms of clinical significance, HIF-2α is a catabolic factor for articular cartilage, and lack of HIF-2α is protective for osteoarthritis development^36^,^37^. We have observed elevated expression of PHD3 in the *Phd2* cKO articular cartilage. This is consistent with our previous report that disruption of *Phd2* in growth plate chondrocytes elevated *Phd3* expression both *in vivo* and *in vitro*^14^. The role of *Phd3* in articular cartilage development and mainanence need to be further investigated.

**Figure 7:**
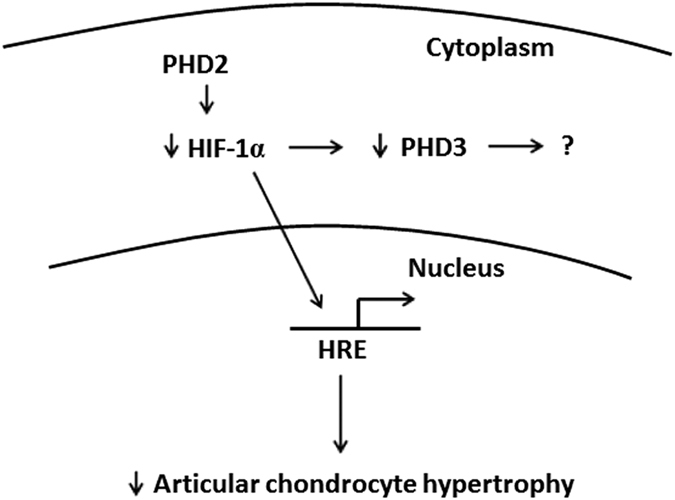
Model of Phd2 regulation of articular cartilage chondrocytes. PHD2 inhibits HIF-1α which in turn inhibits PHD3 expression. In the absence of PHD2, HIF-1α translocates from the cytoplasm to the nucleus, binds to the hypoxia response element (HRE), and activates genes required for chondrocyte hypertrophy. In the presence of PHD2, HIF-1α undergoes prolyl hydroxylation and subsequent proteosomal degradation, and therefore, chondrocyte hypertrophy is reduced.

Based on our findings, we proposed a model for the regulation of *Phd2* on articular cartilage (Fig. 7). PHD2 inhibits HIF-1α, which further inhibits PHD3. Lack of PHD2 promotes HIF-1α signaling and elevates PHD3 expression. HIF-1α translocates from the cytoplasm to the nucleus and binds to the hypoxia response element (HRE) of targeted genes including genes required for chondrocyte hypertrophy. Thus *Phd2* is a key negative regular for articular chondrocyte differentiation (Fig. 7).

## Methods

### Animals

To generate *Phd2* conditional knockout mice specifically in chondrocytes, *Phd2* floxed mice (*Phd2*^*flox/flox*^) were first crossed with *Col2α1-Cre* mice^38^,^39^ to generate Cre positive*, Phd2* loxp-heterozygous mice (*Phd2*^*flox*/+^*; Col2α1-Cre*). *Phd2*^*flox*/+^*; Col2α1-Cre* mice were then backcrossed with *Phd2*^*flox/flox*^ mice, to yield Cre positive, loxp-homozygous (*Phd2*^*flox/flox*^; *Col2α1-Cre*) conditional knockout mice and Cre negative control littermates (*Phd2*^*flox/flox*^*, Phd2*^*flox*/+^). The genetic background of *Phd2*^*flox/flox*^ and *Col2α1-Cre* mice is C57BL/6. Animals were housed according to the approved laboratory conditions in the VMU at VA Loma Linda Healthcare System (Loma Linda, CA).

### Ethics Statement

Animals were housed according to the approved laboratory conditions in the animal facility unit at VA Loma Linda Healthcare System (Loma Linda, CA). All experimental procedures were evaluated and carried out in accordance with the protocols approved by the Institutional Animal Care and Use Committee of the VA Loma Linda Healthcare System. Isoflurane was used for anesthesia, and CO_2_ exposure was used for euthanasia followed by cervical dislocation. All procedures performed followed the ethical guidelines for animal studies.

### Histomorphometry and immunohistochemistry

Four and 12 week old control and cKO mice were sacrificed and the femurs were fixed in 10% formalin for 4 days, decalcified, and processed for paraffin sectioning as previously described^40^. Three comparable sections of the knee joint from each animal were stained with Safranin O and counter stained with Fast Green to visualize the articular cartilage. Measurements of the thickness of the SZ and the MDZ were carried out using the OsteoMeasure software (Osteometrics, Inc. Decatur, GA). OARSI scoring was according to previously described^41^. Immunohistochemistry was performed using the VECTASTAIN ABC-AP kit (AK-5000, Vector Laboratories, Burlingame, CA) as previously described^40^. Antibodies and dilutions of each antibody were listed in Supplemental Table 1. Vector Blue was used as the AP substrate (SK-5300, Vector Laboratories, Burlingame, CA).

### Primary chondrocyte culture and IOX2 treatment

Primary chondrocytes were isolated from articular cartilage of a 3 day old pup knee joint and cultured as previously described^42^. Cells were grown in DMEM/F12 medium containing 10% fetal bovine serum, penicillin (100 U/mL), and streptomycin (100 μg/mL) to 70% confluence followed by 24 hours of serum free (0.1% BSA DMEM/F12) treatment. The cells were then treated with the PHD2 specific inhibitor, IOX2 (Xcess Biosciences, San Diego, CA), at 20 μM or vehicle control (DMSO) for 72 hours.

### shRNA knockdown

MISSION^®^ shRNA Lentiviral Transduction Particles against *Hif-1α* (CCGGTGGATAGCGATATGGTCAATGCTCGAGCATTGACCATATCGCTATCCATTTTTG) and control non-target shRNA were purchased from Sigma-Aldrich (St. Louis, MO). Primary chondrocytes were grown to 25–30% confluence and transduced with *Hif-1α* shRNA or scramble control lentivirus at a multiplicity of infection (MOI) of 10 by adding a pre-made viral particle stock solution (1.4 × 10^7^) into 6-cm culture plates in the presence of 8 μg/ml of polybrene for 2 days, followed by puromycin selection (10 μg/ml) for 3–5 days as previously described^43^. The puromycin selected cells werepropagated and used for gene expression experiments.

### Quantitative RT-PCR

RNA was extracted from cells with Trizol reagent according to the manufacturer’s instructions (Invitrogen, Grand Island, NY). An aliquot of RNA (400 ng) from each sample was reverse-transcribed into cDNA and mRNA levels were followed by quantitative real time PCR as previously described^44^. Relative gene expression values were calculated using the ∆∆CT method with *Ppia* mRNA serving as an internal control^44^. Primer sequences are as following: p57Kip2, forward, 5′-GGAGCAGGACGAGAATCAAG-3′, reverse, 5′-GAAGAAGTCGTTCGCATTGG-3′, cyclin D1, forward, 5′-AATGTACTCTGCTTTGCTGAA-3′, reverse, 5′-ATGAGACCACTAGAGGTCG-3′, tenascin C, forward, 5′-TGACTGCAGCAACCAAGGAC-3′, reverse, 5′-AGTGACCCGCATCTCATTGT-3′, clusterin, forward, 5′-AGAAGGTGAAGATGACCGCA-3′, reverse, 5′-CTTGTACTGCTCTGTCAGCC-3′. Other primer sequences were listed in previous publications^13^,^14^,^45^.

### Statistics

The student’s T-test was used for statistical analysis. *P *<* *0.05 was considered statistically significant. All data were presented as mean ± SEM (standard error of the mean).

## Additional Information

**How to cite this article:** Cheng, S. *et al*. Conditional Deletion of the *Phd2* Gene in Articular Chondrocytes Accelerates Differentiation and Reduces Articular Cartilage Thickness. *Sci. Rep.* **7**, 45408; doi: 10.1038/srep45408 (2017).

**Publisher's note:** Springer Nature remains neutral with regard to jurisdictional claims in published maps and institutional affiliations.

## Supplementary Material

Supplemental Table 1
