# Supplementary material for: Conditional Deletion of the Phd2 Gene in Articular Chondrocytes Accelerates Differentiation and Reduces Articular Cartilage Thickness
Source: Sci Rep. 2017 Mar 28;7:45408. doi: 10.1038/srep45408 (PMC5368651; doi:10.1038/srep45408)
Supplement: Supplemental Table 1 [file srep45408-s1.doc]

**Conditional Deletion of the *Phd2* Gene in Articular Chondrocytes Accelerates Differentiation and Reduces Articular Cartilage Thickness**

Shaohong Cheng^1^, Sheila Pourteymoor^1^, Catrina Alarcon^1^, Subburaman Mohan^1,2, *^

^1^Musculoskeletal Disease Center, Veterans Affairs Loma Linda Healthcare System, 11201 Benton Street, Loma Linda, CA 92357

^2^Department of Medicine, Loma Linda University, Loma Linda, CA 92354

Supplemental Table 1: Antibodies used in immunohistochemistry

| **Peptide/**  **protein target** | **Name of antibody** | **Manufacturer, catalog #, and/or name of individual providing the antibody** | **Species raised in; monoclonal or polyclonal** | **Dilution** |
| --- | --- | --- | --- | --- |
| PHD2 | PHD-2/Egln1(D31E11) Rabbit mAb | Cell signaling  #4835 | Rabbit polyclonal | 1:50 |
| PHD3 | EGLN3/PHD3 Antibody (EG188e/d5) | Novus  NBP1-30440 | Mouse monoclonal | 1:50 |
| HIF-1α | HIF-1 alpha Antibody NB100-134 | Novus  NB100-134 | Rabbit polyclonal | 1:50 |
| HIF-2α | HIF-2 alpha Antibody NB100-122 | Novus  NB100-122 | Rabbit polyclonal | 1:50 |
| HIF-3α | Anti-HIF3 alpha antibody (ab2165) | Abcam  ab2165 | Rabbit polyclonal | 1:30 |
| lubricin | Anti-Lubricin antibody ab28484 | Abcam  ab28484 | Rabbit polyclonal | 1:100 |
| aggrecan | Aggrecan Neo Polyclonal Antibody | Thermo Fisher Scientific PA1-1746 | Rabbit polyclonal | 1:50 |
| COL10 | Anti-Collagen X antibody (ab58632) | Abcam  ab58632 | Rabbit polyclonal | 1:300 |
